# Role of psychiatric intensive care units in preventing long term hospital stays

**DOI:** 10.12669/pjms.42.5.14062

**Published:** 2026-05

**Authors:** Aqsa Shahbaz, Noman Ahmed, Farasat Ali, Ali Madeeh Hashmi

**Affiliations:** 1 Aqsa Shahbaz, MBBS. Postgraduate Resident, Psychiatry, Punjab Institute of Mental Health, Lahore, Pakistan; 2 Noman Ahmed, MBBS, FCPS Psychiatry. Consultant Psychiatrist, Punjab Institute of Mental Health, Lahore, Pakistan; 3 Farasat Ali, MBBS, FCPS, Certificate in Health Professionals Education (CHPE). Associate Professor, Punjab Institute of Mental Health, Lahore, Pakistan; 4 Ali Madeeh Hashmi, MBBS, MD, Diplomate American Board of Psychiatry, MCPS (HPE). Tenured Professor and Chairman, Department of Psychiatry and Behavioral Sciences, King Edward Medical University/Mayo Hospital, Lahore, Pakistan. Professor of Psychiatry, Punjab Institute of Mental Health, Lahore, Pakistan

**Keywords:** Acute psychiatric admission, Hospital length of stay, Inpatient psychiatric care, Psychiatric Intensive Care Unit (PICU), Short-term hospitalization

## Abstract

**Objectives:**

To assess whether psychiatric intensive care unit (PICU) stabilization reduces inpatient length of stay compared with direct admission to wards without PICU support.

**Methodology:**

A retrospective cohort study examined consecutive admissions for acutely disturbed behavior at Punjab Institute of Mental Health, Lahore, from November 1, 2023 to April 30, 2024. Two pathways were compared: PICU admission followed by discharge or transfer to an inpatient unit (PICU group), and direct admission from the Emergency Department to inpatient ward (non PICU group). Demographic and diagnostic variables were recorded; length of stay was compared.

**Results:**

Demographics were similar across units; most patients were male and under thirty-five. Mean length of stay was significantly lower in PICU (6.0 ± 3.09 days) compared to non-PICU (58.9 ± 20.40 days, p < 0.001).

**Conclusion:**

PICU-based stabilization markedly reduced hospitalization duration and prevented long-term admissions.

## INTRODUCTION

Psychiatric Intensive Care units (PICU) are specialty wards for patients suffering from mental illness who need treatment in secure, preferably isolated units because of acutely disturbed behavior.^1^ A PICU is a short-term stabilization unit providing crisis intervention aimed at preventing long term hospitalization by admitting only patients with acutely disturbed behavior posing an immediate risk to self or others.

Acute behavioral disturbance is an emergency condition where a person behaves in a way that might put themselves or others at risk. It is commonly characterized by aggressive or hostile behavior due to fear, paranoia and ideas of persecution or terror, manifesting as absconding behavior, risk of suicide, sexual disinhibition or hyperactivity. This may be due to any underlying mental illness, most commonly bipolar disorder, schizophrenia or substance-induced psychosis. Within a PICU all aspects of the patient’s health, social care needs and risk are assessed and managed by a multi-disciplinary team.^2^

Punjab Institute of Mental Health (PIMH) located in Lahore, Pakistan, a 1510-bed hospital, is one of the largest facilities for psychiatric patients in South Asia established in 1900 as the Government Mental Hospital. As the world progressed towards deinstitutionalization of psychiatric facilities PIMH had its own historical evolution from the medieval practices of confinement to custodial care. Since long it had been operating as a custodial asylum for psychiatric patients.^3^ In a groundbreaking development after becoming a teaching institute in the year 2021, PIMH inaugurated Pakistan’s first Psychiatric Intensive Care Unit (PICU) in September 2023, marking a significant milestone in the nation’s journey towards improvement in mental healthcare provision.^4^

PICUs have been recognized internationally as a crucial part of psychiatric care with established guidelines in countries like UK.^5^,^6^ Furthermore, South Asian and African countries like India and Egypt have studied PICU outcomes in various studies.^7^,^8^ Few studies have highlighted the role of PICU in preventing long term hospitalization^8^,^9^ As PICU is a rather new concept in Pakistan there remains a critical gap in our literature as no such study has been conducted before.

In PIMH as a first of its kind, PICU provides crisis intervention and stabilization services and support to any individual presenting for care in accordance with international guidelines. Its primary aim was to reduce the utilization of hospital emergency rooms, unnecessary inpatient hospitalization and specifically to reduce the length of hospital stay as long-term incarceration of psychiatric patients remains a major issue and often leads to poorer long-term outcomes.^10^

The objective of our study was to determine whether initial stabilization in a psychiatric intensive care unit (PICU) reduces overall hospital length of stay compared with direct admission to inpatient units without a PICU.

## METHODOLOGY

PIMH has five in-patient units (A, B, C, D and E) that function as independent psychiatric wards. All wards have around sixty to hundred beds in a common ward for males and similarly sixty to hundred beds for females each divided in two bays with no isolation rooms. All wards were approximately similar in other respects as well, each having the basic complement of medical officers, nursing staff, a social worker, a consultant and a clinical psychologist. Patients in inpatient wards were admitted directly from Emergency department of PIMH after a 24 hours stay in Emergency.

### Ethical Approval:

This retrospective cohort study was approved by the Institutional Review Board of the Punjab Institute of Mental Health, Lahore. (No. 26977/PIMH, Dated: 18.11.2024).

Twenty psychiatric isolation rooms reserved for acute admission were available in the PICU. Patients in PICU were also admitted through Emergency hence the mode of admission to inpatient wards and PICU was same. Patients were either discharged after management of acutely disturbed behavior or transferred to an inpatient unit for further management after crisis stabilization.

### Treatment programs:

All inpatient units ran on fairly similar lines, with a progress report by a medical officer each morning, once monthly or once quarterly psychotherapy sessions, as required, for most patients, and one grand weekly round by a senior consultant where important diagnostic and treatment decisions were made or confirmed.

The PICU ran through a multidisciplinary approach aimed at rapid stabilization and crisis resolution. An attendant or family member was required to stay with the patient throughout the duration of the hospitalization. Instead of one long consultant ward round every week, daily morning meetings were extended as necessary, so that case presentation, diagnosis and treatment decisions could be decided on daily basis. A post graduate trainee psychiatrist was appointed to monitor the individual patient’s treatment and daily progress. Decisions regarding crisis resolution, expectations regarding progress and an estimated date of discharge from the PICU were discussed at the time of admission and all procedures and progress were actively discussed with family members.

### Data Collection:

Anonymized data from the medical records of all patients admitted for acutely disturbed from November 1, 2023 to April 30, 2024 was collected retrospectively and compared. A consecutive sampling technique was used, whereby all acute admissions through emergency during the study period were included. The data collected from medical records included age, gender, employment status, marital status, diagnosis categorized according to the *Diagnostic and Statistical Manual of Mental Disorders, Fifth Edition* (DSM-5) and length of stay. Patients were routed through one of two pathways: (a) admission to the PICU for early intensive stabilization followed by either discharge or transfer to inpatient unit. (the PICU group) (b) admission to inpatient ward from the Emergency Department without initial PICU stabilization (The non-PICU group).

**Fig.1:**
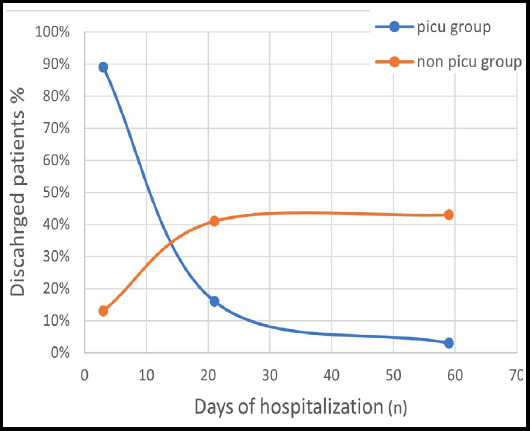
Percentage of patients discharged by time interval and treatment group.

Data were entered and analyzed using Statistical Package for Social Sciences (SPSS) version 26. Stratification was performed for all variables and they were represented as number (n) and percentages (%). Length of stay was represented as Mean ± S.D. Comparisons between groups were made using the chi-square test for categorical variables and the independent t-test for continuous variables. A p-value of less than 0.05 was considered statistically significant.

## RESULTS

Demographic characteristics were broadly comparable in both groups with relatively similar values and none were statistically significant p >0.05. The majority of patients were male (PICU 88 (80%) non-PICU group 96 (85.71%) and under thirty-five years of age (62(56.4%) and 63 (56.25%) respectively). Employment and marital status were similar across groups, with most employed patients earning low wages and married (Table-I).

**Table-I:** Demographic Characteristics.

Characteristic features	Percentage of patients admitted in PICU (n= 110)	Patients admitted directly to inpatient wards (n= 112)
*Gender*	*n (%)*	*n (%)*
Male	88 (80%)	96 (85.71%)
Female	22(20%)	16 (14.29%)
***Age***		
Age: Under 35	62(56.4%)	63 (56.25%)
35 to 60 years	45(40.9%)	48 (42.86%)
Above 60 years	3(2.7%)	1 (0.89%)
***Employment Status***		
Employed	57(51.8%)	50 (44.64%)
Unemployed	53(48.2%)	62 (55.36%)
***Marital Status***		
Married	57(51.8%)	59 (52.68%)
Unmarried	53(48.2%)	53 (47.32%)
***Diagnosis (DSM 5)***		
Schizophrenia	55(50%)	56 (50.0%)
Bipolar disorder	42(38.2%)	41 (36.61%)
Others	13(11.8%)	15 (13.39%)

**Table-II:** Discharge Outcomes

Patient Group	Patients discharged within the study period n (%)	Patients discharged within 6 days n (%)	Patients discharged Within 6 – 21 days n (%)	Patients discharged after 21 days n (%)
Picu group (n=110)	108 (98.2%)	89 (82.4%)	16 (14.8%)	3 (2.8%)
Non picu group (n=112)	61 (54.46%)	8 (13%)	25 (41%)	28 (42.62%)

**Table-III:** Comparison of Length of Stay of discharged patients (t-test).

Variable	PICU group (n=108) Mean ± SD	Non-PICU group (n=61) Mean ± SD	p-value
Length of Stay (days)	6.00 ± 3.09	58.9 ± 20.40	<0.001

Regarding diagnosis, in both groups approximately 55% of admissions were for schizophrenia, with the remainder comprising bipolar disorder (currently manic) and substance-induced psychosis. Other psychiatric conditions, including major depressive disorder, personality disorders, impulse control disorders, accounted for less than 15% of admissions.

A significantly higher proportion of patients in the PICU group were discharged compared to the non-PICU group (98.2% vs 54.5%, p < 0.001). Among PICU admissions, 108 (98%) of patients were discharged during the study period, with 89 (82%) discharged within six days The remaining 21 patients were transferred to an inpatient unit for further management with majority discharged within six to twenty-one days. In contrast, only 61(54.4%) patients in the non-PICU group were discharged. Mean length of stay was significantly lower in PICU (6.0 ± 3.09 days) compared to non-PICU (58.9 ± 20.40 days, p < 0.001).

## DISCUSSION

This study provides a comprehensive evaluation of psychiatric admissions in our hospital over a six months period, with a particular focus on the role of the psychiatric intensive care unit (PICU) in influencing length of stay.

Length of stay emerged as the most significant outcome, with patients stabilized in the PICU demonstrating markedly shorter hospitalizations compared with those admitted directly to general wards. Early discharge expectations appeared to play a key role, facilitating timely mobilization of clinical resources, family support, and patient engagement.^10^-^12^ Discussions with families revealed that prolonged hospitalization was often due to stigma related to psychiatric illness with the common perception of psychiatric illnesses being inevitably deteriorating.^12^ Limited social and financial resources also led families to prefer extended inpatient care.

The PICU’s multidisciplinary approach, incorporating active behavioral management, comprehensive counselling, and continuous family involvement, successfully reduced both hospitalization duration and the risk of institutionalization, a common challenge in long-term psychiatric facilities.^13^-^16^ Transparent communication regarding prognosis and treatment expectations at admission was a distinct strength of the PICU model.^17^ These findings are consistent with international literature demonstrating that specialized intensive psychiatric care models can significantly reduce length of stay while maintaining patient safety.^18^,^19^

Our findings demonstrate that the PICU model serves as a practical implementation of the global initiative to enhance acute psychiatric care services, aligning with recent recommendations to improve access, quality, and outcomes through specialized interventions.^18^-^20^

Our findings underscore the potential of PICUs to optimize clinical outcomes, prevent unnecessarily prolonged admissions, and improve overall patient and family engagement in psychiatric care. The marked reduction in length of stay achieved through PICU stabilization has important implications for healthcare resource allocation, particularly in settings with limited psychiatric beds.^21^,^22^

### Limitations:

First, it was conducted at a single government hospital, the Punjab Institute of Mental Health, which may limit the generalizability of the findings. Most patients and their families belonged to lower socio-economic groups, and outcomes may differ in populations with different socio-economic backgrounds. Second, the retrospective design relies on medical records, which may be subject to incomplete documentation or reporting bias missing variables like duration of illness, illness severity at the time of presentation and number of previous hospitalizations. Finally, the study was conducted over a six-month period, and longer-term trends or seasonal variations were not captured.

## CONCLUSION

As Pakistan moves towards the deinstitutionalization of long-term psychiatric hospitals, the psychiatric intensive care unit (PICU) model offers an effective strategy to reduce prolonged hospital stays and improve treatment outcomes. Implementing PICUs in other large, longstanding psychiatric facilities-such as the Cowasjee Jehangir Institute of Psychiatry, Hyderabad (established 1852), and the Government Mental Hospital, Dadar (established 1939)-could accelerate deinstitutionalization while maintaining patient safety and clinical effectiveness. By providing targeted, intensive care, PICUs have the potential to lower overall healthcare costs, optimize resource utilization, and alleviate the burden on the country’s limited mental health infrastructure. These findings support the broader adoption of the PICU model as a means to enhance both clinical and social outcomes in psychiatric care.

### Recommendations:

Future multi-center, prospective studies with diverse populations are needed to validate and extend these findings.

### Authors’ contribution:

**AS:** Data collection, literature review, drafting of the manuscript, coordination of revisions and responsible for the accuracy of the study.

**NA:** Conception and design of the study, critical revision of the manuscript for important intellectual content, and final approval of the version to be published.

**FA:** Data interpretation, methodological guidance, and critical review of the final draft.

**AMH:** Assisted in data analysis, contributed to the writing and editing of the manuscript, and approved the final version for submission.
